# Middle Rectal Artery Pseudoaneurysm: A Case of Massive Lower Gastrointestinal Bleeding Managed With Coil Embolization

**DOI:** 10.7759/cureus.61573

**Published:** 2024-06-03

**Authors:** Marcus A Spady, Arash Gohari

**Affiliations:** 1 Radiology, Baylor College of Medicine, Houston, USA; 2 Radiology, NYC Health + Hospitals/Lincoln, Bronx, USA; 3 Radiology, Albert Einstein College of Medicine, Bronx, USA

**Keywords:** interventional radiology guided embolization, anal biopsy, perineal condyloma, mra embolization, lower gi bleed, pseudoaneurysm, middle rectal artery

## Abstract

Pseudoaneurysms of the middle rectal artery are rare. When encountered, these have the potential for significant morbidity and mortality due to bleeding and potential rupture. Endovascular embolization is a feasible option in the management of these pseudoaneurysms. The present report describes a case of a 43-year-old male presenting with hemorrhagic shock secondary to lower gastrointestinal bleeding one day after undergoing excision of an external perineal condyloma, incision and drainage of a perirectal abscess, and biopsy of a perianal mass. Angiographic imaging revealed a right middle rectal artery pseudoaneurysm. Selective embolization of the right middle rectal artery and bilateral superior rectal arteries was successfully performed. At the two-week post-embolization follow-up, hemoglobin was stable, and the patient reported normal bowel movements with no episodes of bleeding per rectum.

## Introduction

Lower gastrointestinal (GI) bleeds, defined by bleeding distal to the ligament of Treitz, have an annual incidence of 20.5-27 cases per 100,000 adults with hospital admissions [1,2]. The overall mortality from lower GI bleeds is 2-4% [1]. Most cases of lower GI bleeding will resolve spontaneously; however, there are a few cases that are severe, or “massive”, defined by a drop in hemoglobin (Hgb) by at least 2g/dL and requiring a transfusion of at least two units of packed red blood cells (pRBC) [3,4]. There are several clinical predictors of a severe lower GI bleed including signs of hemodynamic instability (pulse greater than 100 bpm, systolic blood pressure less than 115 mmHg, syncope) and gross blood on rectal examination. Other factors to consider include age, antiplatelet or anticoagulant medication use, comorbid conditions, history of diverticulosis, or history of a prior lower GI bleed [5]. Lower GI bleeds that are severe and that persist despite resuscitative measures require urgent diagnostic and therapeutic intervention.

After evaluating for and ruling out upper GI causes, colonoscopy has been deemed as the first-line management in the diagnosis and treatment of lower GI bleeds [6]. However, colonoscopy is not always a feasible option as it requires bowel preparation in order to facilitate adequate visualization. Flexible sigmoidoscopy and/or anoscopy can be useful in diagnosis if there is suspicion of a distal bleeding source. In hemodynamically unstable patients with significant hemorrhage and non-responsive to resuscitative measures, an alternative option is radiologic identification and endovascular intervention.

The most common causes of lower GI bleeding include diverticulosis, internal hemorrhoids, and ischemic colitis [4]. These top three causes account for over 50% of cases [4]. Other, less frequent, causes include inflammatory bowel disease, post-polypectomy bleeding, colon cancer/polyps, rectal ulcers, vascular ectasia, radiation colitis/proctitis, and other less frequent causes [4]. Here, we present the uncommon case of a 43-year-old male with a massive GI bleed resulting from a middle rectal artery (MRA) pseudoaneurysm after undergoing a series of procedures at the perirectal and perianal regions just days prior.

## Case presentation

A 43-year-old male with HIV (undetectable viral load and a CD4 count >200 cells/mm^3^) presented to the emergency department (ED) for active bleeding per rectum and witnessed orthostatic syncope. Of note, the patient was discharged earlier that day from an outside hospital where one day prior, he underwent excision/debulking of an external perineal condyloma, insertion of setons for two anal fistulas, incision and drainage of a perirectal abscess, and biopsy of a perianal mass extending to the anorectal junction. Initial vital signs upon arrival to the ED were a blood pressure of 100/70 mmHg, a heart rate of 163 bpm, a respiratory rate of 40/minute, and an O2 saturation of 98%. Physical exam was notable for an intact surgical wound at the rectal area and bright red blood per rectum but with no active or pulsatile hemorrhage. The patient was immediately transfused one unit of uncrossed blood, given that he was hypotensive in the setting of a GI bleed. Table 1 shows the initial laboratory results of the patient.

**Table 1:** Initial laboratory results of the patient.

Component	Patient Results	Normal Range
Hemoglobin	5.8 g/dl	Male: 13.2-16.6 g/dl; Female: 11.6-15 g/dl
Hematocrit	18.9 %	Male: 38.3-48.6%; Female: 35.5-44.9%
White blood cells	13.8 x 10^9^/L	3.4-9.6 x 10^9^/L
Platelets	369 x 10^9^/L	Male: 135-317 x 10^9^/L; Female: 157-371 x 10^9^/L

**Figure 1:**
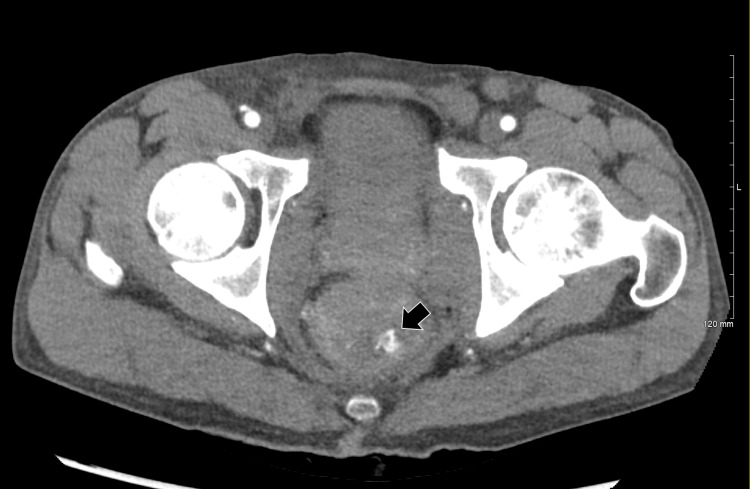
CT image in the axial plane following intravenous administration of iodinated contrast material reveals extravasation (arrow) in the lumen of the sigmoid colon.

The patient was then sent for CT angiography (CTA) of the abdomen and pelvis. The patient returned from the CT scan hypotensive with a mean arterial pressure (MAP) in the 50s, declining mental status, and worsening dyspnea. A massive transfusion protocol was activated. CTA imaging was notable for extravasation of contrast in the sigmoid colon (Figure 1). Interventional radiology (IR) was consulted. Bedside anoscopy revealed clots but a bleeding source could not be identified. Rectal packing was applied. The patient was then transported to the Interventional Radiology (IR) suite for selective mesenteric angiogram and possible embolization. Pre-procedure, the patient was intubated due to labile hemodynamics. The initial selective inferior mesenteric artery (IMA) and superior mesenteric artery (SMA) angiograms were negative for active bleeding. During the procedure, the patient received a total of six units of pRBC and three units of fresh frozen plasma (FFP). The patient was admitted, transferred to the surgical intensive care unit (SICU), and transfused an additional one unit of platelets and one unit of FFP.

The patient remained intubated in the SICU and received multiple transfusions over the following days. The patient underwent a colonoscopy, which was aborted due to the inability to visualize and safely traverse the sigmoid colon, and repeat selective SMA, IMA, and celiac arteriograms, which again showed no evident contrast extravasation. A tagged RBC exam was also performed and demonstrated no evidence of active GI bleeding.

**Figure 2:**
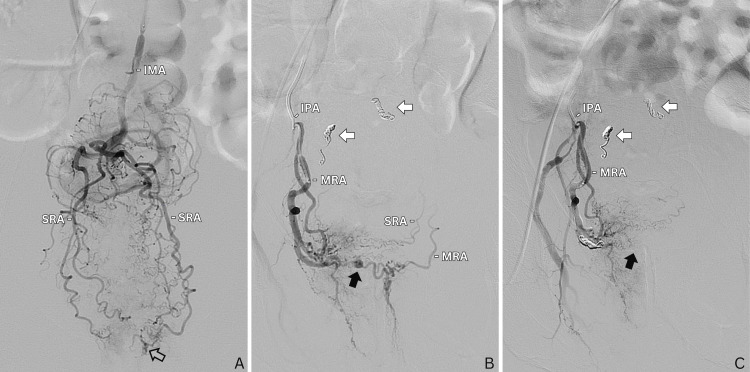
(A) Anteroposterior view of distal IMA injection on DSA demonstrates bilateral superior rectal arteries with subtle extravasation (outlined arrow) from the left SRA. (B) Injection of the MRA, branching off the right IPA, demonstrates a middle rectal artery pseudoaneurysm (black arrow) with retrograde flow into the contralateral MRA and SRA. Coil packing is seen within the right and left superior rectal arteries (white arrows). (C) Post-coiling microcatheter injection of the IPA with no residual filling of the pseudoaneurysm. IMA, inferior mesenteric artery; DSA, digital subtraction angiography; SRA, superior rectal artery; MRA, middle rectal artery; IPA, internal pudendal artery

On the morning of day four, the patient had a large, bloody bowel movement. Once again, the patient was sent to IR for a selective angiogram and embolization. Access was gained through the right common femoral artery with the placement of a 5-french sheath. A selective inferior mesenteric arteriogram was obtained with a 5-french Sos Omni Selective Mariner catheter (AngioDynamics, Latham, New York, United States). From there, a superselective bilateral superior rectal arteriogram was obtained using a 2.8-french Progreat microcatheter (Terumo Medical Corporation, Shibuya City, Tokyo, Japan). Subtle extravasation was seen from the left superior rectal artery (Figure 2A). Bilateral superior rectal arteries were embolized with two 3 mm coils on each side. The decision was made to empirically embolize the contralateral superior rectal artery based on the clinical presentation. A postembolization superselective bilateral superior rectal arteriogram was performed demonstrating adequate occlusion of the superior rectal arteries. The left internal iliac artery was then catheterized. A selective left internal iliac and pudendal artery arteriogram was performed, which demonstrated no active extravasation. Specifically, there was no active extravasation from the identified left MRA on this arteriogram. Subsequently, the right internal iliac artery was catheterized, and an angiogram was performed. Superselective catheterization and angiogram of the right internal pudendal artery were then performed using a 2.8-french Progreat microcatheter (Terumo Medical Corporation). The right MRA was catheterized with the 2.8-french microcatheter. A superselective arteriogram was then performed which revealed a right MRA pseudoaneurysm communicating via collaterals with the left MRA and superior rectal arteries (Figure 2B). The right MRA was embolized with two 3 mm microcoils. The postembolization angiogram demonstrated non-opacification of the pseudoaneurysm with complete occlusion of bilateral superior rectal arteries and right MRA (Figure 2C).

The patient received one unit of pRBC postoperatively. On day five, the patient's Hgb was stable at 8.5 g/dl and he was extubated. The patient reported having some pain with bowel movements that day; however, no frank blood was present in the stool. On day six, the patient was having non-bloody, painless bowel movements. Hgb on this day was stable at 9.5 g/dl. The patient was downgraded from the SICU to the general floor unit. On day eight, while awaiting clearance from physical therapy and infectious disease, the patient decided to leave against medical advice.

Two weeks later, the patient followed up with the surgeon from the outside hospital. During this visit, the patient reported normal bowel movements, with some pain on opening his bowels. Hgb was stable at 9.5 g/dl on the date of this visit.

## Discussion

There are few reports of superior rectal artery pseudoaneurysms causing lower GI hemorrhage and even fewer published reports documenting a MRA pseudoaneurysm [7]. In the appropriate context, given the patient’s history and physical exam findings (i.e., history of rectal surgery, history of anal condyloma, presence of internal hemorrhoids, etc.), when there is suspicion of lower GI bleeding from a distal unidentifiable source, exploration of the MRAs should also be a consideration. CTA can also guide the interventionalist, as in this case where on CTA, contrast was seen in the lumen of the sigmoid colon and arguably at the level of the rectosigmoid junction.

Several reports exist of pseudoaneurysm formation following soft tissue and other organ biopsies [8,9]; however, there are no reports of pseudoaneurysm formation following rectal or anal mass biopsies. Bleeding can, however, be a complication of biopsy of rectal lesions, although it is rarely reported to cause massive GI hemorrhage [10]. Pseudoaneurysms are vascular enlargements that lack a complete arterial wall [11]. Complications of aneurysms include early rupture causing hemorrhage and compression of adjacent structures. Reported sources of pseudoaneurysm formation include trauma, infection, atherosclerosis, and other less frequent causes [11]. The development of the pseudoaneurysm in the presented case was likely secondary to the series of procedures the patient underwent days prior, including incision and drainage of a perirectal abscess. It can be speculated that the pseudoaneurysm also could have arisen as a complication of the local infection itself, termed a mycotic pseudoaneurysm.

Recently, there has been the emergence of rectal artery embolization for the treatment of internal hemorrhoids and there is adequate research that evaluates the feasibility, safety, and efficacy of embolization of these arteries [12]. Supplying the internal hemorrhoids, the most common feeding artery of the hemorrhoidal plexus is the superior rectal artery, which is the terminal branch of the inferior mesenteric artery. In ex-vivo studies, the plexus is found to be rarely supplied by the MRA or inferior rectal arteries [13,14]. However, based on a study that reviewed 250 hemorrhoid embolization procedures, it seems that the MRA can be more commonly involved, with at least unilateral involvement in 33.5% of these cases [12]. This is due to the common presence of anastomoses between the superior rectal artery and MRA. 

The MRA can have variable origins in human anatomy. It typically arises from the anterior division of the internal iliac artery, giving off terminal branches that supply the middle and lower portions of the rectum. In a study by DiDio et al. of 30 cadavers, consisting of 15 males and 15 females, the MRA was seen to be present in 17 cadavers (56.7% of cases), with six of these cadavers presenting with a unilateral MRA [15]. The MRA originated from the internal pudendal artery in 40% of the cadavers, 26.7% from the inferior gluteal artery, and 16.8% directly from the internal iliac artery [15]. In a more recent retrospective study by Bilhim et al. analyzing CTA and digital subtraction angiography (DSA) in 167 male patients with prostate enlargement, MRAs were present in 60 (35.9%) patients, with 20 having a bilateral presence [16]. In their study, anastomoses with the superior rectal and/or other inferior mesenteric arteries were present in 87.5% of cases. They also found that the most common origins of the MRA were the internal pudendal artery followed by the inferior gluteal artery, followed by a common gluteal-pudendal trunk, similar to the aforementioned cadaveric study.

The present case highlights some of the limitations in diagnosing severe lower GI bleeds in an emergency setting. It has been well documented that bleeding from a GI source can be episodic, often resolving without the need for intervention [5,17,18]. Furthermore, bleeding specifically from a pseudoaneurysmal source can be unpredictable and sporadic [19]. In combination, these can be highly difficult sources to identify. The diagnostic capability of selective angiography depends highly on the timing of the procedure, the patient selection, as well as the prowess of the operator. For these reasons, repeated selective angiography may be warranted in cases where the source of bleeding is difficult to identify. As seen in this case, when assessing for the source of a lower GI bleed, selective angiograms of the internal iliac arteries may also be considered to evaluate for all sources of potential bleeding.

## Conclusions

In patients with lower GI bleeds, CTA is a promising diagnostic modality that can be very useful in detecting the source of bleeding and in guiding endoscopic, endovascular, or surgical management. If extravasation of contrast is seen on CTA in the distal sigmoid colon or upper rectum, then this should raise the suspicion that the rectal arteries could be a possible source of the bleeding. If there is concomitant clinical suspicion of a distal source of bleeding, such as in a patient with a recent procedure in the perirectal/perianal regions, then selective angiography of the internal iliac arteries should be performed in conjunction with selective mesenteric angiography. When the source of bleeding is found to be from the rectal arteries, specifically the superior rectal artery or the MRA, embolization of these arteries is effective and safe in the short and mid-term. When done in an emergent setting, such as in this case, the results can be lifesaving. More randomized controlled trials with extended follow-up periods are needed to determine the long-term outcomes of these interventions.
